# Cognitive Performance, as well as Depression, Alcohol Use, and Gender, predict Anti-Retroviral Therapy Adherence in a South African Cohort of People with HIV and Comorbid Major Depressive Disorder

**DOI:** 10.1007/s10461-023-03992-7

**Published:** 2023-01-28

**Authors:** Anna J. Dreyer, Sam Nightingale, Lena S. Andersen, Jasper S. Lee, Hetta Gouse, Steven A. Safren, Conall O’Cleirigh, Kevin G. F. Thomas, John Joska

**Affiliations:** 1 HIV Mental Health Research Unit, Department of Psychiatry and Mental Health, Neuroscience Institute, University of Cape Town, Cape Town, South Africa; 2 Global Health Section, Department of Public Health, University of Copenhagen, Copenhagen, Denmark; 3 Department of Psychology, University of Miami, Miami, FL USA; 4 Department of Psychology, Harvard Medical School, Boston, MA USA; 5 Department of Psychiatry, Massachusetts General Hospital, Boston, MA USA; 6 Department of Psychiatry, Harvard Medical School, Boston, USA; 7 Applied Cognitive Science and Experimental Neuropsychology Team (ACSENT), Department of Psychology, University of Cape Town, Cape Town, South Africa

**Keywords:** Adherence, Antiretroviral therapy, Cognitive impairment, Depression, HIV

## Abstract

Depression and cognitive impairment, which commonly coexist in people with HIV (PWH), have been identified as potential barriers to optimal antiretroviral therapy (ART) adherence. We investigated associations between cognitive performance, depression (as well as other sociodemographic, psychosocial and psychiatric variables) and ART adherence in a South African cohort of PWH with comorbid major depressive disorder (MDD). Cognitive performance and ART adherence were assessed at two time points 8 months apart (*N*_baseline_ = 105, *N*_follow-up_ = 81). Adherence was indicated by self-report, objective measures (Wisepill usage and plasma tenofovir-diphosphate levels), and HIV viral suppression. Mixed-effects regression models examined associations across both time points. Univariate models detected no significant associations between cognitive performance (globally and within-domain) and ART adherence. Multivariate modelling showed increased depression severity (*β* = − 0.54, *p* < 0.001) and problematic alcohol use (*β* = 0.73, *p* = 0.015) were associated with worse adherence as measured subjectively. Being female (*OR* 0.27, *p* = 0.048) and having better global cognitive performance (*OR* 1.83, *p* = 0.043) were associated with better adherence as indicated by viral suppression. This study identifies poor global cognitive performance, as well as depression and problematic alcohol use, as potential barriers to optimal ART adherence in PWH and comorbid MDD. Hence, clinicians could consider assessing for cognitive deficits, depression, and problematic alcohol use, and should endeavour to provide the appropriate support so as to improve adherence.

## Introduction

Antiretroviral therapy (ART) has revolutionised the treatment of people with HIV (PWH). It improves quality of life, reduces HIV-related morbidity and prevents onward transmission of the virus in adherent individuals with suppressed viral loads [1, 2]. Despite these clear and considerable benefits, a significant proportion of PWH are incompletely ART adherent. In South Africa, which has the largest population of PWH (7.8 million) and the largest ART program in the world, incomplete ART adherence is a significant public health concern [3].

One group of PWH identified as being at high risk of incomplete ART adherence are those with depression [4–7]. Nel and Kagee [8] found that South African PWH reporting non-optimal ART adherence were approximately three times more likely to report moderate-to-severe symptoms of depression than those reporting optimum adherence [8].

Depression can also negatively affect cognitive test performance in PWH. The impairing effects of major depressive disorder (MDD) in PWH are typically observed in the domains of motor skills, information processing speed, attention and working memory, learning and memory, and executive function [9–15].

The literature further suggests that there are independent associations between cognitive performance and ART adherence in the general population of PWH [16]. A systematic review [17] summarized this literature and found that impaired cognitive performance (globally and across domains) was associated with worse adherence in 9 of the 11 studies included in sample. The specific domains in which significant associations tend to be present include information processing speed, verbal fluency, attention/working memory, learning and memory, and executive function [16–24].

An important note about that systematic review is that 10 of the 11 studies included in the sample were conducted in the United States, with the lone exception being an Italian study. Clearly, then, few studies investigating this question have been conducted in low- and middle-income countries, such as South Africa. Among these few studies, findings are equivocal and methods may be suboptimal. For instance, whereas a study conducted in Malawi found no relationship between cognitive impairment and adherence [25], another one conducted in Tanzania [26] found that impaired cognitive performance was a strong predictor of suboptimal adherence. However, this latter study used only the Montreal Cognitive Assessment (MoCA), a screening tool, to measure cognitive performance.

In addition to MDD and impaired cognitive performance, other sociodemographic, psychosocial, and psychiatric factors can affect ART adherence. Problematic alcohol use [27–30], increased food insecurity [31–34], younger age [20, 21, 35, 36], fewer years of education [22], and being a woman [18, 37] are associated with non-optimal ART adherence. Of note, however, is that studies conducted in South Africa have found that women are likely to have better ART adherence than men [36, 38].

In the HIV literature, medication adherence is measured using subjective behavioural reports and/or objective ART drug blood levels and pill-taking measures. Subjective reports, which involve reporting if ART was taken as recommended over a specified time interval, are used most commonly [17, 39]. Objective measures, which are considered to be more accurate than self-report [40], include real-time electronic medication monitoring of pill-taking. Various forms of medication event monitoring system (MEMS) technology have been developed to measure adherence [41]. One example of such MEMS technology is the Wisepill box. Each time the pillbox is opened, a signal is transmitted to a web server logging the time and date, thus indicating that medication may have removed from the device at that point [17, 42].

Recent studies have reported on the use of tenofovir diphosphate (TFV-DP) in dried blood spots (DBS) as an objective biomarker of cumulative ART adherence [43]. Due to its long intracellular half-life in red blood cells (17 days), TFV-DP can provide information about average adherence over the preceding 6–8 weeks [44, 45]. Because tenofovir disoproxil fumarate (TDF) forms the backbone of first-line (and some second-line) ART regimens in South Africa, TFV-DP is a particularly useful measure of adherence in this context [46].

Currently, there is no gold standard for measuring ART adherence [41, 47]. Although some consider MEMs to be such a standard [17, 48], others consider biological measures such as TFV-DP to be more accurate [43, 49]. Self-report can accurately measure adherence, although it may tend toward overestimation [39, 50, 51].

Most studies investigating the association between cognitive performance and adherence have used either self-report or MEMS [16, 17, 19, 22, 24, 26]. Of the studies reviewed above, one study measured adherence using pharmacy refill record [18] and another used plasma ART concentrations [25]. To our knowledge, no study has investigated this relationship using TFV-DP in DBS.

Durable high ART adherence (> 80%) is necessary to achieve and maintain viral suppression [52, 53]. Hence, viral suppression is an indication of successful adherence. Of particular interest here is that viral suppression may be independently influenced by cognitive performance, depression, problematic alcohol use, and food insecurity [23, 54–56].

In South Africa (and in other countries with a high burden of HIV), it is critical to identify predictors of ART adherence, especially in vulnerable groups of PWH (such as those with comorbid depression who are incompletely adherent). Such identification could facilitate solutions that can be targeted in a strategic manner to improve adherence and achieve viral suppression. The main aim of this study was to investigate whether, in an incompletely adherent South African cohort of PWH with comorbid MDD, cognitive performance was associated with ART adherence. We also investigated the associations of sociodemographic, psychosocial, and psychiatric factors with ART adherence. Among psychiatric factors, depression was of particular interest.

## Methods

### Setting

Procedures for the current study supplemented those of a larger research program that included a randomised controlled trial of a cognitive-behavioural treatment for ART adherence and depression (CBT-AD; [57–59]).

Participants were recruited at two primary care community clinics in Khayelitsha, a peri-urban community in Cape Town, South Africa. Khayelitsha was established in the mid-1980s under the apartheid regime’s principles of racial segregation. As a consequence of this legacy, today almost all its residents are Black African and it is one of the poorest areas of Cape Town. Most adult residents speak isiXhosa as a first language, fewer than one-third of them have completed high school, and there are high levels of HIV infection, crime, and unemployment [60–64].

### Participants

Participants were 105 PWH with comorbid MDD who had failed first-line ART (i.e., they had already been established as incompletely ART adherent). A subgroup of participants who were not virally suppressed (HIV RNA viral load > 400 copies/mL) at baseline (*n* = 72) were subsequently included in the randomised controlled trial of a CBT-AD [57, 58]. Of those 72, 33 had been assigned to the treatment arm and 39 to a standard-of-care condition.

As part of this study, an additional 33 participants who were not part of the trial were also assigned to the standard-of-care condition. These additional participants were sampled from the same population of PWH as the other participants and were not significantly different from those included in the trial with regard to baseline demographic, psychiatric, or cognitive characteristics. There were also no between-group differences with regard to baseline measures of adherence, expect for the fact that all participants included in the trial were not virally suppressed.

Hence, this study included 33 participants who had been assigned to the CBT-AD treatment and 72 who had been assigned to receive standard-of-care treatment. Of the total sample of 105 participants, 81 (29 in the CBT-AD group, 52 in the standard-of-care group) were assessed again 8 months later.

Inclusion criteria were (a) age ≥ 18 years; (b) HIV-seropositive status (confirmed via medical record); (c) current diagnosis of MDD as measured on the Mini International Neuropsychiatric Interview, Version 7.0 (MINI; [65]); and (d) having failed first-line ART, identified by the community clinic as not having collected ART for > 3 months.

We did not exclude participants with medical and psychiatric co-morbidities and/or other factors that could influence cognitive performance because we wanted our sample to be representative of the clinical population of interest (i.e., PWH with MDD who ART are incompletely adherent). Therefore, the only exclusion criteria were (a) active and untreated severe mental illness (e.g., psychosis or mania) that would interfere with participation, (b) inability or unwillingness to provide informed consent, and (c) lack of sufficient fluency in English or isiXhosa. Participants using antidepressants were eligible even if they met criteria for a current depressive episode; however, they had to have been on a stable antidepressant regimen and dose for at least 2 months.

All participants provided written informed consent. The study protocol was approved by the University of Cape Town (UCT) Faculty of Health Sciences Human Research Ethics Committee and the University of Miami Institutional Review Board.

### Materials

#### Measures of Sociodemographic and Psychosocial Variables

Participants self-reported sociodemographic information (e.g., gender, age, highest level of education, monthly household income, primary language, and employment status). They also completed the Food Insecurity Access Scale (HFIAS; [66]), which measures household food insecurity.

#### Measures of HIV Disease Variables

HIV viral load and current CD4 cell counts were extracted from the medical records. If participants did not have recent (1-month) testing, blood samples for assay were collected using the COBAS AmpliPrep/TaqMan HIV-1 test (range: 20–10,000,000 copies/mL; [67]). ART regimens (i.e., reinitiated on first line, second line, or third line) were also extracted from the participant’s medical record. Participants self-reported whether their nadir CD4 count had ever been below 100 cells/μL.

#### Measures of Psychiatric Variables

The MINI structured diagnostic interview [65] was used to (a) diagnose MDD during screening procedures, so as to establish eligibility for study participation, and (b) screen for the presence of psychiatric disorders that would disqualify individuals from participation. This interview was conducted by a psychiatric nurse and supervised by a clinical psychologist. We used the Alcohol Use Disorders Identification Test (AUDIT; [68]) to identify high-risk alcohol use. High-risk alcohol use was defined using a cut-off score of > 20 on the AUDIT. This is the standard cut-off proposed by the World Health Organization (WHO) and suggests probable alcohol dependence [69]. We used the Hamilton Rating Scale for Depression (HAM-D; [70, 71]) to assess depression severity.

#### Cognitive Assessment

The battery comprised 12 standardized neuropsychological tests, each of which assessed performance in one of seven cognitive domains commonly affected by HIV (Grant, 2008). This battery of tests has been widely used in South African research (e.g., [72, 73]).

Cognitive domains and neuropsychological tests: *Executive functioning*: Color Trails Test 2 (CTT2) and Wisconsin Card Sorting Test (WCST); *Audioverbal learning and memory*: Hopkins Verbal Learning Test-Revised (HVLT-R); *Visuospatial learning and memory:* Brief Visuospatial Memory Test-Revised (BVMT-R); *Verbal fluency*: Category fluency test (animals; fruits and vegetables); *Attention/working memory*: Wechsler Memory Scale-Third Edition (WMS-III) Spatial Span subtest and Wechsler Adult Intelligence Scale-Third Edition (WAIS-III) Digit Span subtest; *Information processing speed*: CTT1, WAIS-III Digit Symbol Coding subtest and WAIS-III Symbol Search; *Motor skills*: Grooved Pegboard Test (GPT) dominant (DH) and nondominant hand (NDH) and Finger Tapping Test DH and NDH.

Tests were administered in either English or isiXhosa, depending on the participant’s preference, by a bilingual neuropsychology technician.

#### Measures of ART Adherence

##### Adherence Questionnaires

We used two self-report adherence questionnaires, labelled here Self-Report Adherence 1 (SRA-1) and Self-Report Adherence 2 (SRA-2). Both ask the respondent to indicate if they took their ART as prescribed over the previous 2 weeks. In SRA-1, participants rated their adherence on a 6-point Likert-type scale with anchor points at *very poor* and *excellen*t. In SRA-2, answers were recorded as a percent of time they were completely adherent, with 10 options ranging from 10 to 100%. Methods of rating adherence using time-frames of longer than 1 week have been shown to be more accurate than 1 week or 3 day time-frames [74].

##### Wisepill

The Wisepill adherence monitor (Wisepill Technologies, Cape Town, South Africa) is an electronic medication monitoring system that consists of a pill box container fitted with a GSM (Global System for Mobile Communication) communication chip. Wisepill was used to measure adherence for participants included in the trial. Wisepill holds approximately 30 large pills or 60 small pills in a two-compartment inner container and is powered by a 1100 mA lithium polymer rechargeable battery (Great Power Battery Ltd, Hong Kong). Each time the device is opened, a cellular signal is sent and recorded in real-time on a web-based server. The data stored there are accessible to researchers via a secure Internet interface. Wisepill technology is a feasible way of monitoring ART adherence objectively and in real time [42, 75].

##### Tenofovir Diphosphate (TFV-DP)

For all participants in the study on ART with TDF as a backbone (e.g., most participants that were reinitiated onto the first line ART regimen were on tenofovir, emtricitabine and efavirenz (TDF/FTC/EFV)), TFV-DP was measured in DBS. Blood was drawn by a nurse into a blood vial. A pipette was then used to extract 50 µl of whole blood from the vial and fill five separate blood spots on the sampling paper (Whatman 903 ProteinSaver card). The card was dried for a minimum of 2 h before being individually packaged in a plastic bag with desiccant. The cards were kept in a refrigerator for a maximum of 1 week, and then stored at – 80 °C. An indirect method for the quantification of TFV-DP in 50 µl human DBS was developed and validated at the Clinical Pharmacokinetic Laboratory in the UCT Division of Clinical Pharmacology. The assay involved a solid phase separation of tenofovir and TFV-DP, enzyme dephosphorylation of TFV-DP to tenofovir, followed by high performance liquid chromatography with tandem mass spectrometry detection of tenofovir [76].

### Procedure

**Fig. 1:**
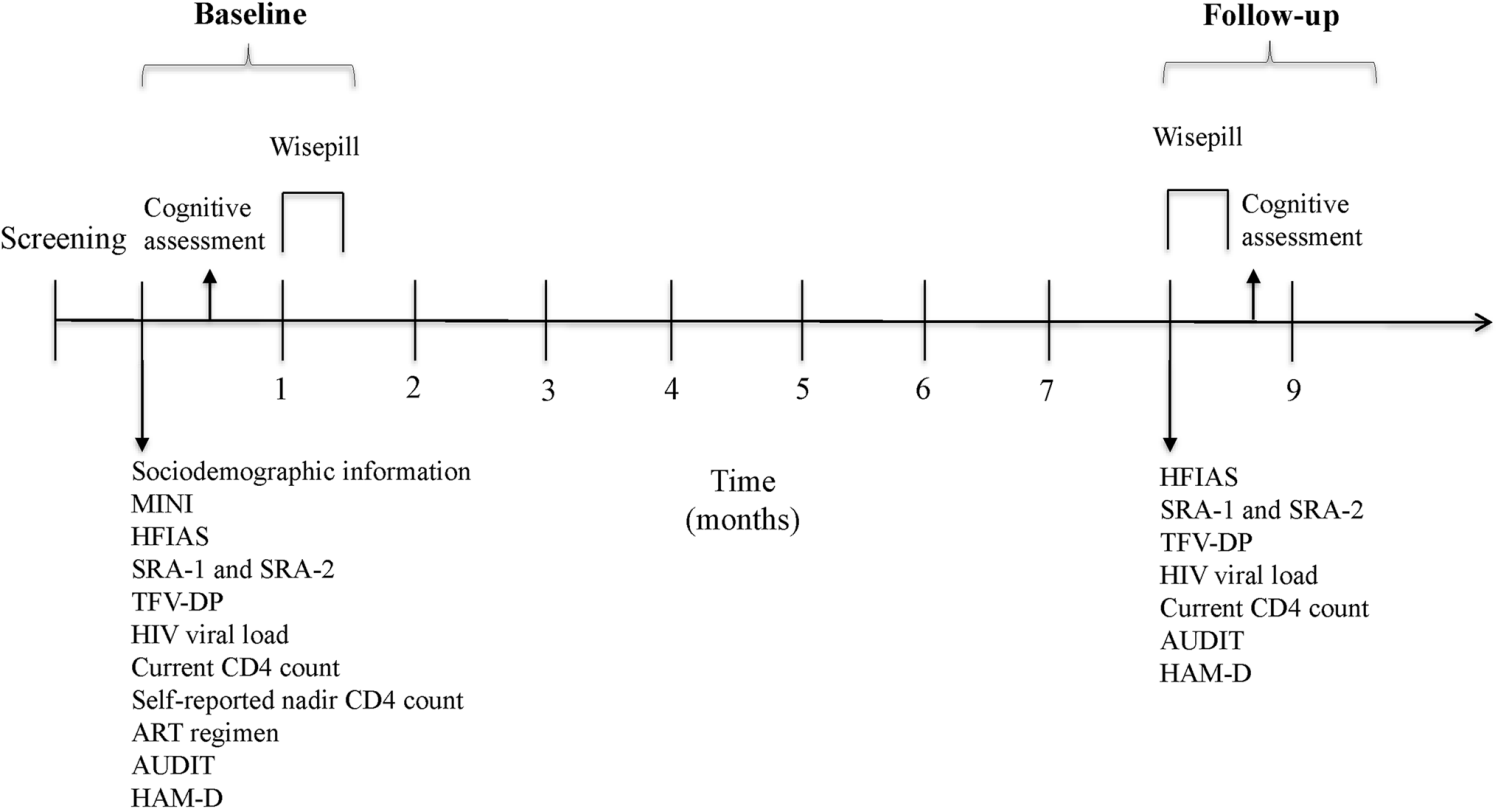
Timeline of study procedures

Figure 1 shows the study timeline and procedures at each time point.

Individuals who remained eligible for study participation after the screening procedures were scheduled for two baseline visits (approximately 2 weeks apart). During the first baseline visit, participants completed measures of sociodemographic, psychosocial, HIV disease, and psychiatric variables. Adherence was measured using the self-report adherence questionnaires and blood was drawn for the DBS sample. During the second baseline visit, participants completed the cognitive assessment. After the set of baseline visits, the 72 participants included in the trial were randomized to receive a CBT-AD treatment or comparison condition described by Joska et al. and Safren et al. [57, 58]. Participants received the Wisepill box at the study visit that they were randomized.

All participants were scheduled for another two follow-up visits 8 months post-baseline. At those visits, they first completed the same measures of psychosocial, HIV disease, psychiatric variables, and ART adherence measures as at baseline and then completed the same cognitive test battery.

### Data Management and Statistical Analysis

#### Deriving Outcome Variables

*Cognitive Outcomes* We derived 15 separate outcome variables from the neuropsychological test data. Outcomes for the domain of *executive functioning* were CTT2 completion time (in seconds) and WCST perseverative errors; for *audioverbal learning and memory*, HVLT-R total across the three immediate recall trials and total on the delayed recall trial; for *visuospatial learning and memory*, BVMT-R total across the immediate recall trials and total on the delayed recall trial; *for verbal fluency*, category fluency test total number of animals and total number of fruits and vegetables named in 1 min; *for attention/working memory*, WMS-III Spatial Span subtest total raw score, WAIS-III Digit Span subtest total raw score, and CTT1 completion time (in seconds); *for information processing speed*, WAIS-III Digit Symbol Coding subtest total raw score and WAIS-III Symbol Search total raw score; and for *motor skills*, GPT DH and NDH completion time (in seconds) and Finger Tapping Test DH and NDH completion time (in seconds).

The raw scores for each of these outcomes were converted to demographically corrected *T*-scores (*M* = 50, *SD* = 10) before statistical analysis of the cognitive data. Normative standards for the tests were based on raw control data collected in previous studies conducted by the UCT HIV Mental Health Research Unit [72, 77, 78]. Data was provided on personal request from co-authors JJ (oral communication, November 2017) and HG (oral communication, June 2018). These data were collected between 2008 and 2016 from healthy community-dwelling individuals (*N* = 233) who presented at the same community clinics in Khayelitsha from which the current sample was recruited. Hence, the control sample and the current sample of HIV seropositive participants were similar across key demographic (age, ethnicity, language, education), psychosocial, and socioeconomic characteristics. In the control data studies, all participants (1) were HIV seronegative, (2) were aged ≥ 18 years, and (3) had ≥ 5 years of formal education. Exclusion criteria for the sample were (1) major psychiatric conditions, (2) neurological disease that could affect brain integrity, (3) lifetime history of head injury resulting in loss of consciousness > 30 min, and (4) current substance use disorder.

We used the control data to calculate demographically corrected *z*-scores, using standard regression-based norming processes. The *z*-scores were then converted to *T*-scores. If participants had *z*-scores greater than 5 SD below the mean, the conversion to a *T*-score resulted in negative *T*-score. In these cases, we assigned a score of zero, the lowest possible *T*-score to maintain the clinical significance of such poor performance. Cognitive data were summarised into domain and global *T*-scores. Domain *T*-scores were calculated by taking the average of *T*-scores of the cognitive outcomes within each domain. Global *T*-score was calculated by taking the average across-domain *T*-scores.

*ART Adherence Outcomes* The outcome variables for ART adherence are SRA-1 and SRA-2, Wisepill, TFV-DP and HIV viral suppression. The raw scores from SRA-1 and SRA-2 were used as outcome variables for self-reported adherence. TFV-DP was recorded as a continuous variable of fmol/punch. A correction factor was applied for the TFV-DP per 50 μL to convert to fmol/3-mm punch, as these units have been used in previous studies (e.g., [46]).

Regarding Wisepill, a dose was counted as “taken” if the pill box was opened within 2 h of the prescribed time. We calculated a percent of doses taken by dividing the number of times the box was opened by the number of prescribed doses over a 2-week period, following each of the time points (i.e., baseline and follow-up). Some participants had their follow-up cognitive assessment closer to the 12-month follow-up in the larger trial. For these participants we recorded percentage Wisepill use over the 2 weeks preceding the 12-month follow-up visit. Wisepill data was only available for the participants included in the trial (*n* = 72).

HIV viral suppression was defined at plasma viral load < 400 copies/ml, which was the threshold of viral breakthrough in a similar population of PWH in Cape Town [46].

#### Inferential Analyses

We used R version 4.1.2 (2021-11-01) and RStudio version 2021.09.0 to complete all inferential analyses, with the threshold for statistical significance set at α = 0.05.

Before beginning the analyses that allowed investigation of the study’s major aims, we (a) calculated sample descriptive statistics for baseline values of the relevant variables, (b) calculated sample descriptive statistics for ART adherence outcomes at baseline and at follow-up, and (c) conducted a principal component analysis (PCA) with a varimax rotation to create composite variables from the measures of ART adherence. Specifically, we ran a PCA using SRA-1, SRA-2, Wisepill, and TFV-DP to determine the components the measures loaded onto so that we could combine the measures to create composite ART adherence variables. For an item to be retained in a component, it needed to have a factor loading > 0.4 and no higher loading on another factor. Creating a composite of adherence measures is recommended to more accurately measure adherence and, in this case, helped determine whether the various measures of ART adherence were measuring the same construct [41, 50]. Viral suppression was retained as its own outcome variable because of its value as a commonly used and important clinical indicator.

Our first major analysis investigated univariate associations between cognitive performance and ART adherence at both time points. Here, linear mixed-effects regression models featured as predictors each of the domain *T*-scores and global *T*-score and as outcomes each ART adherence composite variable and viral suppression. We also included a random intercept for participants to account for multiple observations (i.e., that the same individual completed each measure twice, at baseline and at follow-up). The utility of this random intercept was to inform the model that it should expect there to be multiple responses per participant, and that these responses will depend on each participant’s baseline level. This enabled us to pool together data from both time points. Analyses included data from both time points to increase statistical power.

Our second major analysis used linear multivariate mixed effects regression modelling to determine the measured variables most strongly associated with incomplete ART adherence at both time points. Again, data from both time points were included analyses to increase statistical power. There were three separate models: one for each ART adherence composite variable, and one for HIV viral suppression. In the fully specified models, we entered the following as fixed effects: sex (male versus female), age, education (years completed), HFIAS score (as an estimate of food insecurity), AUDIT score (above versus below the cut-off score [≥ 20] for high-risk alcohol use), global *T*-score (as an estimate of cognitive performance), and HAM-D score (as an estimate of depression symptomatology). We also included a random intercept for participants to account for multiple observations (i.e., that the same individual completed each measure twice, at baseline and at follow-up). Each model was built using a backwards stepwise approach, starting with the fully specified model and sequentially dropping variables that were not contributing to the model. Each time a variable was removed, the new model was compared to the previous model using a chi-square test to ensure that the removal did not affect the model negatively.

We investigated statistical assumptions and outliers for all regression-based analyses. Influential outliers were investigated using Cook’s distance. We investigated any point > 4/*n*, where *n* is the number of observations. Outliers were removed from the analyses if the model improved without them. An Intraclass Correlation Coefficient (ICC) > 0.10 was considered high and indicated a multilevel analysis was relevant [79].

## Results

### Descriptive Statistics

Table 1 presents the sample’s demographic and clinical characteristics. Most participants were women, which is typical of the South African PWH population [80]. isiXhosa was the primary language of most participants (93%). The sample’s median monthly household income was 1600 ZAR (100 USD). Most participants (85%) had not completed high school (in South Africa, this is 12 years capped by a major exit examination), 89% were unemployed, and 68% had experienced severe food insecurity. Participants had all been identified by the community clinic as failing first line ART and therefore were either reinitiated on first line or placed on second- or third line ART regimens. All participants had a primary diagnosis of current MDD and the average HAM-D score was within the ‘severe depression’ range [81] at baseline.

**Table 1:** Demographic and clinical variables at baseline: descriptive statistics (N = 105)

Variables	*M* (*SD*)	*f* (*%*)
Sociodemographic and psychosocial		
Sex (female)		76 (72.38%)
Age (years)	39.79 (9.14)	
Education (years completed)	9.30 (2.45)	
Monthly household income (ZAR)	1600 (0–2900) ^a^	
HFIAS ^b^	12.74 (6.90)	
HIV disease		
Log10 HIV viral load	3.56 (1.44)	
Current absolute CD4	248.49 (209.38)	
ART regimens		
Reinitiated		56 (53.85%)
Second		47 (45.19%)
Third		1 (0.96%)
Self-reported nadir CD4 count < 100 cells/ml		68 (64.76%)
Psychiatric		
HAM-D score	25.63 (7.10)	
High risk alcohol use ^c^		30 (28.85%)
Cognitive Performance by Domain (*T*-score)		
Motor skills	46.53 (11.32)	
Information Processing Speed	46.36 (9.83)	
Verbal Fluency	50.40 (7.86)	
Attention and Working Memory	43.50 (9.87)	
Audioverbal Learning and Memory	50.00 (11.10)	
Visuospatial Learning and Memory	48.17 (7.53)	
Executive Functioning	43.17 (9.96)	
Global Cognitive Performance (*T*-score)	46.87 (6.34)	

^a^Median (interquartile range)

^b^Higher score indicates greater food insecurity

^c^High risk alcohol use indicated if Alcohol Use Disorders Identification Test (AUDIT) score > 20. *M* (*SD*) = mean (standard deviation); ZAR = South African Rands; HFIAS = Household Food Insecurity Access Scale; ART = antiretroviral therapy; HAM-D = Hamilton Rating Scale for Depression

Table 2 presents descriptive statistics for the ART adherence outcomes at both baseline and follow-up.

**Table 2:** ART Adherence measures at Baseline and Follow-up: Descriptive Statistics

Variables	*n*	Baseline		*n*	Follow-up	
		*M* (*SD*)	*f* (*%*)		*M* (*SD*)	*f* (*%*)
SRA-1	103	2.33 (1.42)		80	3.60 (1.33)	
SRA-2 (%)	104	60.54 (30.58)		80	80.07 (20.50)	
Wisepill (%)	70	67.70 (31.67)		51	51.09 (38.78)	
TFV-DP (fmol/punch)	48	634.75 (521.88)		39	669.20 (526.64)	
HIV viral suppression ^a^	105		25 (23.81%)	81		40 (49.38%)

*SRA* self-report adherence; *TFV-DP* Tenofovir diphosphate. *M* (*SD*) mean (standard deviation)

^a^HIV RNA viral load < 400 copies/mL

### ART Adherence Composite Variables

The PCA resulted in a two-factor solution that explained 82% of the variance. Based on this analysis, all four ART adherence measures were retained. The two self-report variables loaded onto one component (Eigenvalue = 1.875; factor loadings were SRA-1 = 0.964 and SRA-2 = 0.956), which explained 47% of the variance. Wisepill and TFV-DP loaded onto another component (Eigenvalue = 1.406; factor loadings were Wisepill = 0.823 and TFV-DP = 0.850), which explained 35% of the variance.

Hence, we created two separate composite variables for the ART adherence measures: The first is the Self-Report Adherence Composite (composed of SRA-1 and SRA-2), and the second is the Objective Adherence Composite (composed of Wisepill and TFV-DP). We calculated values for each of these composite variables by multiplying the values for each of the original ART adherence outcome variables by the factor loadings and adding them together.

### Univariate Associations Between Cognitive Performance and ART Adherence

Analyses detected no significant associations between any of the cognitive performance predictor variables (each of the domain *T*-scores and the global *T*-score) and any of the ART adherence outcome variables (Self-Report Adherence Composite, Objective Adherence Composite, viral suppression; see Table 3).

**Table 3:** Univariate associations between cognitive performance and ART adherence

Predictor variables	Outcome variables											
	Objective Adherence Composite (*n* = 96)				Self-Report Adherence Composite (*n* = 105)				HIV Viral suppression (*n* = 104)			
	est	CI	t	p	est	CI	t	p	OR	CI	z	p
Domain *T*-scores												
Motor skills	− 0.00	− 0.02 to 0.01	− 0.27	0.785	0.01	− 0.02 to 0.03	0.68	0.500	1.03	0.99 to 1.08	1.53	.125
Information Processing Speed	− 0.00	− 0.02 to 0.01	− 0.50	0.617	0.00	− 0.02 to 0.03	0.31	0.756	1.04	0.99 to 1.09	1.63	.103
Verbal Fluency	− 0.00	− 0.02 to 0.02	− 0.04	0.967	0.01	− 0.02 to 0.05	0.80	0.427	1.03	0.97 to 1.09	1.00	.319
Attention and Working Memory	0.01	− 0.02 to 0.03	0.54	0.586	0.02	− 0.02 to 0.05	0.89	0.376	1.49	0.97 to 2.28	1.82	.069
Audioverbal Learning and Memory	− 0.01	− 0.03 to 0.00	− 1.53	0.129	0.02	− 0.01 to 0.05	1.48	0.141	1.03	0.99 to 1.08	1.45	.148
Visuospatial Learning and Memory	− 0.00	− 0.02 to 0.01	− 0.34	0.738	0.01	− 0.02 to 0.03	0.68	0.495	1.02	0.98 to 1.05	0.88	.382
Executive Functioning	− 0.02	− 0.03 to − 0.00	**− **1.98	0.050^a^	0.02	− 0.01 to 0.05	1.35	0.179	1.01	0.97 to 1.05	0.53	.598
Global *T*-score	− 0.09	− 0.26 to 0.07	− 1.10	0.272	0.20	− 0.08 to 0.47	1.38	0.168	1.08	0.99 to 1.16	1.82	.068

*Est* estimate, *CI* confidence interval

^a^Data from one participant were removed from the analyses because the value was an influential outlier (Cook’s D = 0.06)

### Multivariate Modelling of ART Adherence

#### Self-Report Adherence Composite

The final model included the HAM-D and AUDIT variables as fixed effects. For every one unit increase in HAM-D score, this adherence composite score decreased by 0.54 points (CI: − 0.78, − 0.30; *t* = − 4.43; *p* < 0.001; *r* = − 0.27) on average across both time points. For participants with AUDIT scores ≥ 20 (i.e., those reporting high-risk alcohol use), this adherence composite score was 0.73 points lower (CI: − 1.31, − 0.15; *t* = − 2.47; *p* = 0.015; *r* = − 0.21), on average across both time points, compared to participants with AUDIT scores < 20.

For this final model, the marginal *R*^2^ was 0.13, indicating that these fixed effects explained 13% of the variance in the outcome.

We used several measures to determine the model’s robustness. The ICC was 0.24, which supports the use of a linear mixed model. The conditional *R*^2^ was 0.34, which also confirms the importance of having a random effect for participant in the model (i.e., 34% of the variance in the outcome was explained by this random effect). Another indication of the model’s robustness is that the final model (AIC = 730.69) was significantly better than the null model (AIC = 751.00; *χ*^2^ = 24.31; *p* < 0.001).

#### Objective Adherence Composite

The final model included only age as the fixed effect, which was not statistically significantly associated with this adherence outcome (estimate = 0.16; CI: − 0.01, 0.32; *t* = 1.87; *p* = 0.064, *r* = 0.16). The marginal *R*^2^ was 0.02, indicating that this fixed effect explained 2% of the variance in the outcome.

Again, we used several measures to determine the model’s robustness. The ICC was 0.16, which supports the use of a linear mixed model. The conditional *R*^2^ was 0.18, which also confirms the importance of having a random effect for participant in the model (i.e., 18% of the variance in the outcome was explained by this random effect). The final model (AIC = 473.50) was not significantly better than the null model (AIC = 472.11; *χ*^2^ = 3.39; *p* = 0.066).

#### HIV Viral Suppression

Results for this model (a binary logistic multivariate mixed effects regression model) are reported in Table 4. The final model included sex, age, HAM-D score, and global *T*-score as fixed effects.

**Table 4:** HIV viral suppression across both time points: final binary mixed effects regression model

Fixed Effect	Odds ratio	*CI*	*z*	*p*
Sex (male versus female)	0.27	0.07–0.99	− 1.98	0.048*
Age (years)	1.79	0.99–3.24	1.94	0.053
HAM-D score	0.61	0.36–1.02	− 1.87	0.062
Global *T*-score	1.83	1.02–3.29	2.03	0.043*

*HAM-D* Hamilton Rating Scale for Depression; *CI* confidence interval

*p < .05

Analyses indicated that both sex and global *T*-score were statistically significant. On average across both time points, female participants were 0.27 times more likely than male participants to be virally suppressed. For every one-unit increase in global *T*-score, the odds of the participant being virally suppressed increased by 1.83.

For this final model, the marginal *R*^2^ was 0.15, indicating that the fixed effects explained 15% of the variance in the outcome.

The ICC for this model was 0.45, which supports the use of a mixed effects model. The conditional *R*^2^ was 0.53, which also confirms the importance of having a random effect for participant in the model (i.e., 53% of the variance in the outcome was explained by this random effect). Another indication of the model’s robustness is that the final model (AIC = 230.33) was significantly better than the null model (AIC = 240.02; *χ*^2^ = 17.69; *p* = 0.001).

## Discussion

The main aim of this study was to investigate associations between cognitive performance and ART adherence in a South African cohort of PWH with comorbid MDD. We also investigated associations between ART adherence and a set of sociodemographic, psychosocial, and psychiatric variables. Among the psychiatric variables, depression was of primary interest.

Univariate analyses indicated that neither global nor within-domain cognitive performance were significantly associated with any ART adherence outcome. Multivariate models showed that global cognitive performance was not significantly associated with ART adherence (measured both subjectively and objectively). However, when controlling for depression severity and education level, better global cognitive performance (and being female) was significantly associated with greater probability of being virally suppressed.

One might have expected, based on results reported by previous studies, that poor cognitive performance in individual cognitive domains would have been significantly associated with worse ART adherence, regardless of whether the outcome was measured subjectively or objectively [17–21, 23]. For instance, impaired executive functioning can make medication management more challenging, and impaired learning and memory can result in difficulties remembering to take medication [17, 19].

One explanation for our non-significant univariate results might involve sample characteristics: Participants in our sample were (at baseline, at least) diagnosed with MDD, whereas participants in previous studies that found significant associations were not reported as experiencing such psychiatric illness. The presence of depression may modify relations between cognitive performance and ART adherence given that there are independent associations between depression and cognitive performance [9–15] and between depression and ART adherence [4–8].

This speculation is supported by results from the multivariate models. Global cognitive performance was significantly associated with viral suppression in a multivariate model that controlled for depression severity and education level. In other words, when not controlling for these factors (as in the univariate models), the presence of MDD and low levels of education (on average, the sample had only completed 9 years of education) may have masked the significance of the association between overall cognitive performance and viral suppression. However, when the statistical controls are put in place, the significant association is revealed.

Considering our finding that global cognitive performance was significantly associated with ART adherence as indicated by viral suppression but that it was not significantly associated with the other measures of ART adherence (i.e., by either the other objective or the subjective indices), it may be that the measures that we used in fact measure slightly different aspects of adherence within the same general construct. More specifically, viral suppression is more properly considered an outcome of successful ART adherence, rather than a measure of adherence [3, 82]. Also, viral suppression likely reflects cumulative optimal adherence over a relatively long timeframe [83, 84], whereas our other measures provide more current indications of adherence (we took Wisepill reports over a 2-week period and TFV-DP and self-report outcomes at two discrete time points). In other words, what our results imply is that poor cognitive performance may be associated with historical poor adherence, as indicated by viral suppression, but may not be associated with recent incomplete adherence (as measured by self-report, Wisepill, and TFV-DP).

Increased depression and problematic alcohol use were significant predictors of worse self-reported ART adherence. This finding is consistent with previous research showing that, independently, depression [4–8] and problematic alcohol use [27–30, 85] predict incomplete adherence in PWH.

Being female was significantly associated with increased chances of being virally suppressed. This finding is consistent with other South African studies suggesting that women have better ART adherence than men [36, 38, 86]. In South Africa, men with HIV are less likely to access and remain linked to HIV care [87, 88]. Several mechanisms may explain this gender disparity, including the prioritisation of maternal and child health services in the South African public health systems and gender differences in health-seeking behaviour [88].

We acknowledge the following limitations of the study design. First, the relatively small sample size means the study may have been underpowered to detect potentially significant associations between, for instance, within-domain cognitive performance and ART adherence, or between any of the predictors and objectively measured ART adherence. Second, because viral suppression assesses HIV treatment efficacy and is not simply an outcome of successful ART adherence, non-suppression may indicate viral resistance to ART rather than incomplete adherence [40]. Third, we cannot infer a causal direction in the relationship between adherence and cognitive performance. Although we imply above that poor cognitive performance leads to poor adherence, the opposite relationship between cognition and adherence (i.e., incomplete ART adherence resulting in worse HIV disease which in turn causes more cognitive impairment) has been reported by others [19].

## Conclusion

The results from our mixed-effects regression models indicated that, in this incompletely adherent sample of PWH with comorbid MDD, lower levels of cognitive performance were associated with HIV viral suppression. Men were also less likely than women to be virally suppressed. Finally, participants with more severe depression and high-risk alcohol use self-reported worse ART adherence, although this pattern of data was not replicated in objective markers of adherence.

Because successful ART adherence is crucial to improving the lives of PWH, preventing HIV-related mortality, and reducing new infections, it is vital to identify barriers to optimal adherence in groups of PWH identified as being at high risk for incomplete adherence. This endeavour is particularly important in South Africa, which has the largest population of PWH and the largest ART program in the world [3]. Current adherence interventions in our setting are primarily aimed at addressing depressive symptoms (e.g., [57]) and problematic alcohol use (e.g., [89, 90])—none target cognitive underperformance. Interventions aimed at improving adherence and achieving successful HIV suppression in vulnerable cohorts of PWH should explore cognitive screening and practical forms of cognitive rehabilitation.

## Data Availability

The datasets generated during and/or analysed during the current study are available from the corresponding author on reasonable request.

## Code Availability (Software Application or Custom Code)

The R code used to analyse the data for this study is available from the corresponding author on reasonable request.
